# Assessment in the context of problem-based learning

**DOI:** 10.1007/s10459-019-09909-1

**Published:** 2019-10-02

**Authors:** Cees P. M. van der Vleuten, Lambert W. T. Schuwirth

**Affiliations:** 1 School of Health Professions Education, Faculty of Health, Medicine and Life Sciences, Maastricht University, P.O. Box 616, 6200 MD Maastricht, The Netherlands; 2 Prideaux Centre for Research in Health Professions Education, College of Medicine and Public Health, Flinders University, Sturt Road, Bedford Park, SA 5042 Australia

**Keywords:** Assessment, Competency-based medical education, Constructive alignment, Problem-based learning, Programmatic assessment, Progress test

## Abstract

Arguably, constructive alignment has been the major challenge for assessment in the context of problem-based learning (PBL). PBL focuses on promoting abilities such as clinical reasoning, team skills and metacognition. PBL also aims to foster self-directed learning and deep learning as opposed to rote learning. This has incentivized researchers in assessment to find possible solutions. Originally, these solutions were sought in developing the right instruments to measure these PBL-related skills. The search for these instruments has been accelerated by the emergence of competency-based education. With competency-based education assessment moved away from purely standardized testing, relying more heavily on professional judgment of complex skills. Valuable lessons have been learned that are directly relevant for assessment in PBL. Later, solutions were sought in the development of new assessment strategies, initially again with individual instruments such as progress testing, but later through a more holistic approach to the assessment program as a whole. Programmatic assessment is such an integral approach to assessment. It focuses on optimizing learning through assessment, while at the same gathering rich information that can be used for rigorous decision-making about learner progression. Programmatic assessment comes very close to achieving the desired constructive alignment with PBL, but its wide adoption—just like PBL—will take many years ahead of us.

## Introduction

Since its inception, problem-based learning (PBL) has conquered the world (Donner and Bickley 1993). Its history is described in a number of publications (Schmidt 2012; Servant-Miklos 2019). What started in the mid-sixties at McMaster University as a radical break from lecture-based education (Barrows and Tamblyn 1980), turned out to be a successful didactic strategy which has since been increasingly copied by other schools. Originally, PBL had a high ideological identity. This meant that it was defined as a process with defined steps which had to be adhered to when practicing ‘true PBL’. Only later did it become clear that PBL aligned with insights and theories from educational and cognitive psychological research (Norman and Schmidt 1992; Dolmans et al. 2005; Neville 2009). PBL emphasized the need for problem-solving to train clinical reasoning (Norman 1988) and for long it had been assumed that the method itself would teach students to become generic clinical reasoners. This assumption fueled a long line of research on what constitutes (clinical) reasoning expertise (Schmidt and Rikers 2007). Through this, PBL became more scientifically grounded over the years.

Nowadays, the original ideological approach to PBL has calmed down and it can have many different manifestations. So, when schools claim to be using PBL it is not always clear what that exactly entails.

In our view that are some essential characteristics:

1. The use of engaging tasks or problems as a starting point for learning
2. Self-directed and self-regulated learning
3. Working in groups of learners tackling these tasks
4. The role of the teachers as a facilitator of this process

Many of these characteristics can be explained also by current insights from further educational theory. The use of meaningful tasks is an example of a whole task approach as is promoted by educational design theories (Merrienboer and Kirschner 2007). Collaborative learning theories underpin the use and the conditions for effective group learning (Johnson et al. 2007), while giving autonomy to learners in their learning process resonates with theories of motivation and self-determination (Deci and Ryan 2008). The widespread use of PBL is undoubtedly promoted by the scientific underpinning of the approach.

This leaves the question how to design assessment of learner achievements in the context of PBL? Constructive alignment has been suggested as a concept that expresses the extent to which the intended goals of the training program align with the overt and unexpected goals of the the assessment as espoused and experienced by all stakeholders (learners, staff and organization) (Biggs 1996). If there is a mismatch between the two, the assessment impact typically overrides the intended learning approach.

The dominant educational practice in assessment is a summative, modular approach, particularly assessing the more cognitive aspects. Learners typically progress from module to module, passing or failing standardized tests along the way. Unfortunately, many PBL schools use this approach as well, which logically leads to constructive misalignment in many cases.

To better understand this constructive misalignment, we find it helpful to identify two major frictions around assessment in a PBL context. The first is that PBL is assumed to promote more than purely the development of knowledge and skills. Such other abilities related not only to clinical reasoning and clinical decision-making, but also to more domain independent abilities such as communication, collaboration professionalism, etcetera. Despite the ongoing challenges in clearly defining these domain independent abilities they are generally seen as important and incorporated visibly in all the major competency frameworks (Anonymous 2000; Frank and Danoff 2007; GMC 2013). The perceived friction between what was generally assessed and what was aspired by PBL education approaches has led to many attempts to design more appropriate methods of assessment.

The second friction lies in the contradiction of requiring the learners to self-regulate their learning on the one hand, but at the same time they have to successfully pas set of teacher-led assessments or tests. Again, the concept of self-regulation of learning in the educational setting is not undisputed. For example, the ability of students to successfully self assess and subsequently direct their own learning is seriously doubted (Eva et al. 2004). Yet, there seems to be more agreement that after graduation doctors should be able to be lifelong learners and for this require having developed self-assessment and self-regulated learning ability. This conceptual friction has led to a quest of assessment strategies that better fit to PBL.

In this paper, we will discuss each of these frictions in more detail and some of the assessment developments that arose from this search for better constructive alignment. In doing so, we will also discuss other major developments in education and in assessment that are not related to PBL itself, but which have a major impact on how to deal with constructive alignment in PBL.

## The quest for instrumentation

Clearly, PBL is aimed at promoting clinical reasoning, which logically led to the desire to develop instruments for the assessment of clinical reasoning, and subsequently to a vast amount of research and development in this area. A comprehensive overview on the developments on assessment of clinical reasoning is from Schuwirth et al. (2019).

Within the assessment literature, this started in the sixties with the use of paper simulations of patient problems (McGuire and Babbott 1967; McCarthy and Gonnella 1967). They were called Patient Management Problems (PMPs). A patient’s initial complaint was presented, and the learner had to navigate their way through the problem to arrive at the solutions. Each action taken was scored and these scores were considered to be an indication of a person’s clinical reasoning ability.

Several, counterintuitive, measurement problems with the method were found. First, experts did not agree on the optimal pathway through the simulation and assigned different credits to each decision. In other words, when different experts were presented with the same problem, they suggested different solution pathways.

Second, it was discovered that the scores of individual learners across patient problems was very low, in the order of 0.1–0.2. It became clear that clinical reasoning could not be measured as a generic and knowledge-independent trait. This was a first indication of what later has been called the problem of content specificity (Eva 2003). Content specificity was subsequently found to be innate to almost all assessment measurement. In order to arrive at a reproducible score in all assessment measurements, considerable sampling needs to be done across sources of variance; aspects that have a possible impact on the score such as content (problems, cases, items, orals, stations, etc.), assessors, (Van der Vleuten and Schuwirth 2005). The corollary of this that given that assessment time is limited, there is a need to be efficient with sampling. One of the developments were assessment methods with short scenarios or vignettes which were less complex, such as key-feature approach testing (Page et al. 1995) and or extended-matching items (Case and Swanson 1993). However, these instruments seemed to focus mainly on the outcome of the clinical reasoning process, the clinical decision making. The assessment of the reasoning process itself still remained a Holy Grail. Therefore, the search continued and some more specific clinical *reasoning* instruments were developed later, based on insights from the clinical expertise literature. One example is the Script Concordance Test (SCT) in which an ill-defined patient scenario unfolds itself and the learner has to indicate probabilities of their hypothesis of the problem (Lubarsky et al. 2011). Another format was an oral that also mimicked the PBL learning process, the so-called Triple Jump Exercise (Westmorland and Parsons 1995). It started with the presentation of a case in an oral setting (jump 1), some time for self-study on the case by the learner (jump 2) and a report of the finding in a next oral session (jump 3). The method was quite original but never has gained much popularity.

One of the currently proposed reasons why clinical decision making was easier to assess than clinical reasoning is an ontological difference: clinical decision making is a process that typically leads to one or a few defensibly correct answers whereas clinical reasoning is a process that is more unpredictable or complex and there can lead to multiple good answers depending on the situation (Durning et al. 2010). Both aspects are probably equally important for any competent clinician but the fundamental difference has fundamental implications for their assessment. If good clinical decision-making predictably leads to correct answers, it can typically be tested with structured and standardised assessments. That is why the key feature approach to assessment and extended matching items have been found to be valid (Case and Swanson 1993; Bordage et al. 1995). When the required outcome is unpredictable and there are multiple good answers depending on the situation the assessment cannot be predefined and has to happen in the here and now. One example of this challenge is illustrated by the concerns around script concordance tests, where the stimulus—what the question asks—is divergent in nature but the scoring is convergent and hence does not sit well with the complexity of clinical reasoning (Lineberry et al. 2013). This has led to a renewed interest in researching the role of human judgment in the assessment of clinical reasoning (Govaerts et al. 2012; Govaerts et al. 2011; Gingerich et al. 2014). Still, the common mechanism for assessing clinical decision making and clinical reasoning is to use authentic but efficient clinical tasks, usually in the form of patient scenario’s, as a stimulus for obtaining responses.

There can be many variations to do this in an assessment practice. Schuwirth et al. conclude: “Finally, because there are so many ways to assess clinical reasoning, and no single measure is the best measure, the choice is really yours.” (Schuwirth et al. 2019, p. 413) and will depend on your resources, the appeal of the approach and the potential learning value of the method of choice. Many modern approaches to clinical decision-making use patient scenarios in efficient question formats (Case and Swanson 2002). By using efficient methods for clinical reasoning and decision-making part of the alignment friction may be alleviated.

However, PBL was also assumed to promote other abilities than knowledge and skills, such as collaboration, communication and regulated learning ability and professionalism. Therefore, initiatives were undertaken to develop instruments for the assessment of these abilities. At McMaster University, where PBL started, initially tutor-based assessment of the learners was used. Actually, during the first years these were the only assessments. Later, other, more standardized assessments were added for various reasons. One of those was that the tutor evaluations did not predict licensing exam performance (Keane et al. 1996). One can question whether this inability to predict performance on a licensing exam is an indication that the assumption of good self-regulated learning being sufficient to predict the development of competence is incorrect or whether the early implementation of purely human judgement-based assessment was still immature. Since that time, much has been learned about using human judgement in assessment, partly from the literature on heuristics and biases (Plous 1993) and from naturalistic decision-making (Gigerenzer and Goldstein 1996) and in the context of assessment of medical competence (Govaerts et al. 2011, 2013a; Gingerich 2015). At Maastricht University for instance, the second university to adopt PBL, the assessment of professional behavior received a prominent place (Van Luijk et al. 2000; Van Mook et al. 2009). These assessments were based on a judgement and narrative feedback from the tutor and peers combined with a self-assessment on behavior pertaining to group work around the task, in relation to others in the group and to oneself. Essentially, these were early examples of the use of professional judgment to assess more complex abilities. A salient distinction between both developments is that in the initial years at McMaster the tutor evaluations were used as the predictor for medical competence as a whole, whereas the Maastricht development entailed a much closer alignment between purpose of assessment and process. Yet, the downside of this was a persistence of the compartmentalisation of the assessment of competence.

Another development in education, competency-based medical education (CBME), proposed a more integrative view on competence, in which all types of abilities were expected to interact with each other. So for assessment, this required a more integrative view. In the CBME literaturea ‘competency’ is generally defined the integration of knowledge, skills and attitudes to fulfil a complex professional task (Albanese et al. 2008), which instigated a major orientation shift in educational thinking. CBME challenged education to define the outcomes of education as: “What is it that learners after completing the training program are able to do?” Different organizations developed competency frameworks (Anonymous 2000; Frank and Danoff 2007; GMC 2013). These frameworks were constructed with wide stakeholder input and were strongly influenced by the expected needs of future healthcare. Competency frameworks have had a profound impact on structuring curricula, but they also influenced the assessment developments and their research. The commonality across these frameworks that they emphasize complex abilities, such as communication, collaboration, professionalism, health advocacy, systems-based practice, etcetera, more strongly. These abilities are important because they were found predictive for success and failure in the labor market (Papadakis et al. 2005; Semeijn et al. 2006; Van Mook et al. 2012). Complex abilities cannot be easily defined, though and neither can they be easily trained in a short course ending with an exam. These competencies usually require vertical learning lines in a curriculum and develop longitudinally. Through its increase in popularity CBME challenged the traditional measurement perspective of assessment and stimulated developers and researchers to start ‘assessing the unmeasurable’. it is generally help that these complex abilities cannot be measured at one point in time but can only be assessed through professional judgments of *habitual* performance in more or less *authentic* educational or clinical settings. This means that they can hardly be captured in a simple checklist and when tried, the assessment is trivialized (Van der Vleuten et al. 2010). Thus, the assessment literature moved towards the top of Miller’s pyramid (Miller 1990): the assessment of performance using unstandardized measures that strongly rely on more subjective sources of information (Kogan et al. 2009). This did not negate that every student is entitled to a fair and equitable outcome of the assessment, but not to exactly the same process to reach at outcome. This conceptual shift in thinking was essential in addressing the friction between self-regulation and self-direction of learning in PBL in the traditional standardised assessment.

Another major consequence of the attention to CBME is the issue of longitudinality. Looking at growth across time is a fundamental challenge for our classical approach of a modularised assessment system. What originally were early and perhaps marginal attempts in PBL schools to assess complex abilities gained considerable attention through the shift towards CBME.

We are still in the midst of understanding the consequences of these developments. One of the obvious implications is that in workplace-based assessment the observation and scoring have to happen simultaneously. This is different to, for instance, written examinations where a whole series of subjective judgements (what is the curriculum, what is the blueprint what topics to questions, what items to produce, what standards to set?) precedes the collection of performance data (which can be even done by a computer program). This requirement of real-time observation and scoring required considerably more assessment literacy from the assessor and could not simply be solved by more elaborate rubrics (Popham 2009; Valentine and Schuwirth 2019).

What is further evident, is that more attention is given to assessment from an education perspective, rather than from the dominant discourse around psychometrics in standardized assessment technology, i.e. in the first three layers of the pyramid (Schuwirth and Ash 2013). The learner and the utility of assessment to inform learning became more central (Kogan et al. 2017). Watling and Ginsburg posited that we are on a discovery journey on understanding the right “alchemy” between assessment and learning (Watling and Ginsburg 2019). Some of the recent insights include that medicine is a relatively poor feedback environment (Watling et al. 2013b) in which it differs substantially and surprisingly from other high-performance domains. Feedback itself has been extensively studied (Van de Ridder et al. 2015; Bing-You et al. 2017). A fundamental finding is that feedback must stem from a credible source (Watling et al. 2012), and it must be logical, coherent and plausible as well as constructive. Logically, poorly given feedback will have limited—or even negative—impact. Another finding showed that in highly summative settings, learners are less inclined to engage with feedback (Harrison et al. 2016). Perhaps the most important implication is that scores and grades have considerable limitations as information conveyers. Qualitative and narrative information have much more meaning than scores, particularly when complex abilities are being assessed (Ginsburg et al. 2013). For instance, Ginsburg et al. showed more measurement information to be found in narrative data than in quantitative data (Ginsburg et al. 2017) and that residents were well able to read between the lines even when the feedback was generally positively framed (Ginsburg et al. 2015). The ample attention to feedback and learning has produced concrete and helpful suggestions around what to do in assessment, what not to do and what we don’t yet know (Lefroy et al. 2015). In recent years, the attention has shifted from a process of giving feedback to the importance of trusted social relationships (Ramani et al. 2019). Ideally, feedback is a dialogue either in action, based on direct observation of a clinical event, or on action, over a longer period of time (Van der Vleuten and Verhoeven 2013).

The same holds for self-directed learning; self-directed learning requires educational scaffolding, for example through an ongoing dialogue with a trusted person. The literature on mentoring is shows early positive effects (Driessen and Overeem 2013).

In all, CBME has forced the assessment literature into exploring and developing better work-based assessment and to rethink our strategies around assessment and learning (Govaerts et al. 2013b). It clearly is about the right alchemy. Assessment should have an obvious learning function through providing the learner with meaningful feedback. Feedback use is to be scaffolded with feedback follow-up or through dialogues with entrusted persons with a growth mindset. The culture of a clinical setting or a department is over overriding importance as it conveys the strongest messages to the learner about what is expected and what is sanctioned (Watling et al. 2013a; Ramani et al. 2017). Creating an assessment culture with a growth mindset in which assessment information is used to promote better learning and growth and development. Although these concepts seem to be reasonably developed in the literature, the actual practical implementation is not always easy. At the coalface, there are still fundamental conceptions of so-called naïve beliefs that contravene the literature (Vosniadou 1994); the non-domain specific abilities are labelled ‘soft’ skills and are deemed more peripheral aspects of medical competence and often too hard to assess, and the notion of lifelong learning seems to be at odds with the notion of licensing and credentialling at a certain point in time.

Nevertheless, the interplay between learning, assessment of complex abilities and self-directed learning are elements which are central to the CBME movement and also pivotal for assessment in PBL.

### The quest for assessment strategies

PBL seeks to foster a deep learning strategy, focused on conceptual understanding. Assessment strategies to promote such learning strategies haves been on the agenda since the beginning of PBL. Probably, the Triple Jump Exercise mentioned earlier is an example of an approach to promote deeper understanding by mimicking the PBL learning cycle.

Another alternative assessment strategy that has a long history in PBL is progress testing (Schuwirth and van der Vleuten 2012). A progress test is a comprehensive written test—often with vignette based items—that represents the end objectives of the curriculum, comparable to a final examination, so in fact contains relevant questions out of the whole domain of functional medical knowledge. That test is administered to all the learners from all years in a program, but of course with different standards per year class. The test is repeated a number of times per year, each with new questions but with the same content blueprint. The results on the individual tests are combined to produce growth curves and performance predictions. This form of testing started in 1977 in Maastricht. The main purpose was to avoid test-directed studying. It is very difficult to specifically prepare for a progress test since anything might be asked. But, if a learner studies regularly in the PBL system most likely sufficient growth will occur automatically. Therefore, there is no need to cram or to memorize; actually it is a counter-productive preparation strategy (Van Til 1998). Due to its longitudinal nature—and especially in context where several medical schools collaborate and jointly produce progress tests, such as Germany, the UK, Italy and Brazil, the progress test, provides a wealth of information for individual students about their own learning and for schools to compare their performance with other schools

Longitudinal assessment is also assumed to be a better predictor of future performance. From a PBL philosophy of educating lifelong learners, this is important. Progress test predicts for instance performance on licensing examinations (Norman et al. 2010). When the University of McMaster adopted progress testing it was a valuable addition to existing tutor evaluations. This kind knowledge testing without the side effect of test-directed studying and that is predictive for licensure performance fitted their PBL approach hand-in-glove. From a strategic perspective, the interesting question is what in existing assessment programs may be replaced with progress testing. There are schools that rely exclusively on progress testing in the cognitive domain (Ricketts et al. 2009) and it is easily conceivable how many resources would be saved if no other knowledge exams were needed. Progress testing as a strategy of assessment has gained a definite place in the context of PBL. It reinforces many of the intentions of PBL and has proved itself practically and empirically.

A wider assessment strategy, is programmatic assessment. Programmatic assessment looks strategically to the assessment program as a whole (Schuwirth and Van der Vleuten 2011; Van der Vleuten et al. 2012). The ground rules in programmatic assessment are:

- Every (part of an) assessment is but a data-point
- Every data-point is optimized for learning by giving meaningful feedback to the learner
- Pass/fail decisions are not given on a single data-point
- There is a mix of methods of assessment
- The choice of method depends on the educational justification for using that method
- The distinction between summative and formative is replaced by a continuum of stakes
- Stake and decision-making learner progress are proportionally related to the stakes
- Assessment information is triangulated across data-points towards a competency framework
- High-stakes decisions (promotion, graduation) are made in competence committees
- Intermediate decisions are made with the purpose of informing the learner on their progress
- Learners have a recurrent learning meetings with (faculty) mentors using a self-analysis of all assessment data

Programmatic assessment requires an integral design of assessment in a program. Deliberate choices are made for methods of assessment, each chosen to maximally align with the intended learning goals. Learning tasks themselves may also be considered as contributing assessment tasks. For example, writing a critical appraisal on a clinical problem as part of an EBM track can be a data-point. From a conceptual point the assessment aligns maximally with the educational objectives. Any individual data point is never used to make high-stakes decisions (Van der Vleuten and Schuwirth 2005). That way, by taking out the summative “sting” out of each individual assessment, learners may concentrate on a learning orientation rather than trying to game of summative assessment. Self-directed learning is promoted through regular data-driven self-assessment and planning of learning, reinforced and supported by a trusted person that follows the learner in time (usually across years of training). Data points need to be rich in nature. When quantitative, the richness lies usually in feedback reports on subdomains and comparative information is given to a refence group. When qualitative, the richness lies in the quality of the narrative being provided. The use of professional judgment (by faculty, coworkers, peers or patients) and direct observation are strongly promoted and supported by capacity building processes in programmatic assessment. Decision-making becomes robust by triangulating and aggregating information across data-points. Since the information across data points is a combination of quantitative and qualitative data, decision making cannot be algorithmic or statistical, and human judgment is indispensable. Any high-stakes decision is rendered robust by using independent decision committees that arrive at their decisions by using rich information and reaching consensus (Hauer et al. 2016), when needed through iterative consultative processes. Procedural strategies derived from qualitative research are used to build the trustworthiness of the competence committee decision (Driessen et al. 2005; Van der Vleuten et al. 2010). For example, the committee will elaborately deliberate and motivate when there is doubt about the decision to be made. Programmatic assessment has been implemented in a number of undergraduate (Dannefer and Henson 2007; Wilkinson et al. 2011; Heeneman et al. 2015; Bok et al. 2013; Jamieson et al. 2017) and postgraduate settings (Chan and Sherbino 2015; Hauff et al. 2014). Its practical proof of concept has been produced, and there is considerable research ongoing on programmatic assessment [see Van der Vleuten et al. (in press) for a summary]. However, it will take many more years to fully scientifically underpin this integrative and knowledge of doodle approach to assessment. The biggest challenge is securing sufficient buy-in from faculty and students. Programmatic assessment requires a different mindset from the people involved. It is an escape from the traditional summative assessment paradigm. The process of moving towards programmatic assessment from traditional assessment practice as a deep conceptual mind shift bears striking similarities to the challenges PBL faced when it sought to supplement or replace traditional lecture-based curricula. As such, one could argue there is even a constructive alignment with respect to the change process.

## Conclusion

Assessment in the context of PBL is driven by the need for constructive alignment between intentions of PBL and assessment. The classic summative paradigm with end-of-unit examinations does not really fit well to PBL. Although an initial search for instruments relevant for PBL may have produced some promising developments, it has become clear that no single instrument can unveil the whole picture. This has been a general conclusion in the assessment literature for any given training program (Van der Vleuten et al. 2010). Constructive alignment is best achieved through an integrative approach to assessment (Norcini et al. 2018; Eva et al. 2016) and for this to be attained a breach with the traditional summative approach is required. Programmatic assessment is such an example. In essence, it is similar to the idea of progress testing, but it incorporates all competencies and the assessment program as a whole. Like with all innovations, it will take time before it will be adopted more widely in the various PBL training programs. This is not unexpected as PBL itself took many years before wide-scale adoption occurred and many years of research to better understand it. This will also be the case with a more holistic view on assessment where assessment not only drives learning but learning drives assessment. Just like in PBL we will see many different manifestations or “hybrids” in system wide approaches to assessment. However, slowly and many years after the start of PBL, assessment has an answer to the needs of PBL.
